# Gastaut-Geschwind Syndrome, Faciobrachial Dystonic Seizure, and Autoimmune Limbic Encephalitis

**DOI:** 10.1155/2018/3835819

**Published:** 2018-12-03

**Authors:** Ori-Michael J. Benhamou, Mohammad Tavakkoli, Hande Okan, Mitchell Nobler

**Affiliations:** Department of Psychiatry, Westchester Medical Center, USA

## Abstract

Here we report a case of a 55-year-old male who had presented with recent falls and behavioral changes, including a heightened religious preoccupation, hypergraphia, and paranoid ideations. He was initially treated for psychosis but soon exhibited absence-like seizures, which were consistent with faciobrachial dystonic seizures. Workup for underlying infectious, immunodeficiency, and autoimmune causes revealed antibodies towards the leucine-rich glioma inactivated subunit of the voltage-gated potassium complex. The patient was treated with steroids and intravenous immune globulin with symptomatic relief. In retrospect, the patient met criteria for Gastaut-Geschwind (GG) syndrome, with notable features of hypergraphia and hyperreligiosity. This case illustrates how the GG syndromal pattern contributes to the suspicion of autoimmune limbic encephalitis and may expedite diagnosis and prevent the accumulation of disability.

## 1. Introduction

Faciobrachial Dystonic Seizure (FBDS) is a rare form of epilepsy characterized by frequent brief seizures, which primarily affect the arm and face. It has been described as the pathognomonic semiology for autoimmune limbic encephalitis (ALE) [1]. ALE is most commonly caused by nonparaneoplastic autoimmune antibodies directed towards the intracellular antigen, glutamic acid decarboxylase (GAD65), or the extracellular antigens, contactin-associated-protein-like 2 (CASPR2) and leucine-rich glioma inactivated-1 (LGI1), both members of the voltage-gated potassium channel complex (VGKC) [2]. The FBDS phenomenon has been specifically associated with anti-LGI-1 antibodies [1]. In most cases, FBDS has been described to precede behavioral and psychiatric symptoms of ALE [1, 3].

Gastaut-Geschwind (GG) syndrome encompasses a set of behavioral changes that are pronounced in some patients diagnosed with temporal lobe epilepsy [4–6]. The syndrome manifests with personality changes and behavioral changes that include increased irritability, hyperreligiosity, an exaggerated philosophical concern, atypical or decreased sexuality, interpersonal “stickiness”, circumstantial thought process, and compulsive documentation [7]. There is controversy as to the existence of such a syndrome as it correlates to seizure disorders [8].

## 2. Case Report

We describe a case of a 55-year-old man who presented with six months of progressive cognitive decline, multiple recent falls of unknown etiology, and behavioral changes. The falls had resulted in widespread bruising, which the patient attributed to “arthritis”. His behavioral changes notably included a heightened religious preoccupation, in which he had joined four new churches and would spend hours every day walking along highways between these institutions as well as a synagogue. He had registered several of the pastors as his healthcare proxies. He would spend further hours of his day documenting his thoughts and reading philosophical writings. In addition, he had become convinced that his parents were “out to get me”.

On initial presentation, the patient's delusions were limited to paranoia and he denied perceptual disturbances. He was irritable and impulsive, attempting to elope on several occasions from the emergency department. His thought process had become “sticky”, in which he would adhere to or ruminate over specific ideas and give long-winded circumstantial responses to simple questions. He became convinced that he was in the hospital for his arthritis and that the hospital staff was intentionally trying to manipulate his brain. Upon admission, he called several pastors to visit and quickly amassed a collection of religious and philosophical books as well as notebooks, in which he would document his thoughts and the behaviors of staff on the unit.

Interestingly, from the second day on the inpatient unit, he exhibited intermittent “absence-like” seizures, consisting of an abrupt onset of staring, accompanied by abnormal stereotypic movements of the left arm and facial contraction. During these episodes, the left side of the patient's face would spasm—mouth being pulled upwards and backwards and left eye blepharospasm—with a concurrent flexion and raising of the left arm and immediate resolution to prior position within five seconds. These events occurred periodically and irregularly several times each day and were associated with transient anterograde amnesia. The patient had a complete lack of insight into these occurrences to the point of carrying a phrase to completion despite experiencing an attack partway through the sentence.

**Figure 1:**
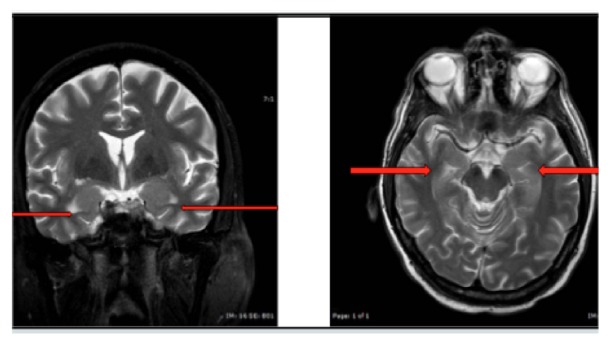
MRI brain demonstrating bilateral mesial temporal lobe hyperintensities (red arrows), right greater than left, without volume loss: (left panel) T2-weighted Coronal section; (right panel) T2-weighted with FLAIR axial section.

Other than superficial bruising, physical examination was unremarkable. Vital signs were stable other than a fluctuant pulse rate that would periodically rise to sinus tachycardia in the 130's. Routine laboratory values were unexceptional, other than mild hyponatremia (Na = 131-133). Surface Video EEG was negative for epileptiform activity despite capturing several of the clinical episodes noted above. Brain MRI without contrast showed bilateral T2-hyperintensities (R>L) in the parahippocampal gyri without volume loss in the temporal mesial structures (see Figure 1). To target his mood symptoms and seizure activity, he was initially started on valproic acid and titrated to a therapeutic dose over the course of one week; however, his seizure activity was refractory and increased in frequency with worsening of mental status and memory deficits. His treatment was thus augmented with risperidone and titrated to 2 mg twice daily over the ensuing two weeks, which resulted in a modest reduction in paranoia and ruminations; however, his cognition continued to decline, with a progression of negative symptoms to near catatonia and his seizures persisted. CSF obtained from a lumbar puncture was sent to an outside laboratory for serology on an autoimmune panel and confirmed the presence of antibodies towards the LGI1 portion of the VGKC complex and no other autoimmune antibody including anti-CASPR2, another anti-VGKC antibody. He was transferred to the neurology service and diagnosed with anti-LGI1 ALE. He was promptly treated with intravenous methylprednisolone 1 g daily and intravenous immune globulin 400 mg/kg/day, with instructions to continue on oral prednisone, as per recommendations from the literature [9–11], leading to improvement of his disorganization and psychiatric symptoms and to a substantial decrease in his dystonic movements within days but a slower resolution of his cognitive impairment over the following month.

## 3. Discussion

This case report illustrates a patient whose age, gender, neuroimaging [12], hyponatremia, and dystonia that is refractory to antiepileptic medications [13] are consistent with other documented cases of anti-LGI1 ALE in the literature. However, the specific constellation of psychiatric symptoms and their chronological antecedence to FBDS has not been well described. The patient's symptom cluster of hypergraphia, exaggerated religious fixation, viscous thought process, paranoia, memory deficits, and irritability is reminiscent of GG syndrome, a somewhat controversial disorder described in the mid-late 20th century as a behavioral manifestation of temporolimbic dysfunction [14]. Notably, the patient's neuroimaging demonstrated a larger lesion in the right medial temporal lobe, in accordance with Hoffman's findings of the GG behavioral pattern in right temporal lobe stroke victims [15]. Hypergraphia is an unusual feature of GG syndrome that has previously been reported as specific to nondominant right hemispheric temporal lobe lesions [16] or donepezil-induced mania [17]. Another distinctive characteristic of GG syndrome, hyperreligiosity, has also been reported to be specific to right temporal lobe lesions [18, 19].

Although patients frequently present to psychiatric institutions with symptoms of paranoia, cognitive disturbances, and altered thought processes, hypergraphia and intensified religious preoccupation are relatively uncommon and suggest an interictal personality disorder, even without reported seizure activity. Taken together, the GG syndromal pattern may contribute to the suspicion of ALE, rooted in anti-LGI1 antibodies localized to the nondominant temporal lobe, prior to the start of FBDS, expediting the diagnosis and preventing the accumulation of disability.
